# Effect of Dietary Supplementation of Biochars on Growth Performance, Bone Mineralization, Footpad Health, Lymphoid Organs Weight, Antibody Titers of Newcastle Disease and Infectious Bronchitis Disease in Broiler Chicks

**DOI:** 10.3390/vetsci12070680

**Published:** 2025-07-18

**Authors:** Raheel Pervaiz, Riaz Mustafa, Umar Farooq, Waseem Abbas, Muhammad Farooq Khalid, Abdur Rehman, Munawar Hussain, Muhammad Muzammil Riaz, Asfa Fatima, Muhammad Aziz ur Rahman

**Affiliations:** 1 Institute of Animal and Dairy Sciences, University of Agriculture Faisalabad, Faisalabad 38000, Pakistan; kraheel162@gmail.com (R.P.); waseem.abbas@uaf.edu.pk (W.A.); muzammilriazfsd@gmail.com (M.M.R.); asfa3396@gmail.com (A.F.); 2 Constituent College Toba Tek Singh, University of Agriculture Faisalabad, Faisalabad 38000, Pakistan; drriaz@uaf.edu.pk (R.M.); u.farooq@uaf.edu.pk (U.F.); farooq325@uaf.edu.pk (M.F.K.); 3 Department of Animal Sciences, Faculty of Agriculture, Sargodha University, Sargodha 40100, Pakistan; abdur.rehman@uos.edu.pk; 4 Sind Feed and Allied Products, Karachi 75600, Pakistan; munawar.manais@gmail.com

**Keywords:** corncob biochar, digestibility, growth performance, litter score, poultry production, wheat straw

## Abstract

This study evaluated the effect of biochars on growth performance, nutrient digestibility, carcass yield, bone mineralization, litter quality and footpad lesions in broilers. Eight hundred day-old chicks were randomly divided into four treatments, 10 replicates per treatment (20 birds/replicate) for 35 days. Treatments were basal diet (control), a control diet with corncob (CC) biochar (1%), a control diet with wheat straw (WS) biochar (1%) and a control diet with sugarcane bagasse (SCB) biochar (1%). Body weight gain (BWG), feed intake (FI) and feed conversion ratio (FCR) were recorded weekly. Nutrient digestibility, bone mineralization and carcass parameters were determined on the 21st and 35th days, while footpad lesions and litter quality were also assessed. The results revealed significant improvement (*p* < 0.05) in FI, BWG and FCR with supplementation. Nutrient digestibility was higher (*p* < 0.05) in the SCB biochar group. Tibia calcium and phosphorus levels were enhanced (*p* < 0.05) in the WS and SCB biochar groups, respectively. Footpad lesions were significantly lower (*p* < 0.05) in the CC biochar group, while litter quality was improved (*p* < 0.05) in the WS biochar group. Lymphoid organ relative weight results revealed that spleen weight was not affected by biochar supplementation in diet (*p* > 0.05), while dietary supplementation of CS and WS biochar in the diet resulted in the highest relative weights of thymus and bursa (*p* < 0.05). However, dietary supplementation of WS, SC and SCB biochar supplementation had affected positively the log value of the ND virus and IBV titers in birds. Overall, dietary supplementation of 1% biochars enhances growth performance, bone mineralization, footpad health immunity and litter quality in broilers.

## Simple Summary

Use of agriculture by-products in animals feed as a feed additive not only ensures sustainable livestock production but also reduces the negative environmental impact of agriculture by-products. Our study provides evidence that dietary supplementation of 1% biochars improved body weight gain by up to 4.7%, reduced feed conversion ratio by 6.7% and enhanced tibia calcium and phosphorus content. Additionally, it improved footpad health, litter quality and titer against Newcastle disease and infectious bronchitis disease in broilers.

## 1. Introduction

Litter management is considered a critical step to improve broilers’ productivity. For optimum quality of litter, it is essential to maintain the moisture content of litter between 20 and 30% [1]. Higher moisture levels of litter resulted in compromised litter quality. It causes a higher incidence of dermatological conditions like footpad dermatitis [2,3] and increased ammonia excretion. All these factors have a negative impact on birds’ health and ultimately result in decreased growth performance [4,5]. It emphasizes the use of such types of feed ingredients that ameliorate the litter quality by minimizing the excretion of ammonia and improve the health of the birds. Biochar is a low-cost carbon-rich material for reduced ammonia excretion [6,7]. Biochar is not only known for improving the litter quality but also enhancing the health and performance of the poultry birds. Biochar, a carbon-rich product, is obtained via the decomposition of organic matter at high temperature and an anoxic environment by the process of pyrolysis [8] and contains 50% carbon content [9]. Racek et al. [10] state that biochar is produced from a thermochemical variation of biomass in an oxygen-limited environment at 400 °C.

Previous studies reported that biochar is also a good source of important minerals such as calcium and phosphorus and results in improved mineralization of bones [11], growth performance and litter quality [12,13]. However, studies also reported that addition of biochar may negatively influence the performance of the broilers due to the presence of arsenic [14]. Nevertheless, this negative effect is mostly overcome by the addition of biochar in the diet of poultry in limited quantity and supplementation of various feed additives like phytase in biochar-containing diet [15].

The other reason for using phytase in poultry diet is the presence of phytate in the diet. It has been reported that poultry diet is normally a cereal-based diet, and phosphorus in cereals is present as phytate, rendering it indigestible for poultry due to their limited endogenous phytase enzyme [16]. Phytate has anti-nutritional effects like reducing the digestion of nutrients, increasing the endogenous loss of nutrients and reducing production performance [17]. To boost the production performance by increasing phosphorus bioavailability, the phytase enzyme is used [17]. Numerous studies have indicated that supplementation of poultry feed with phytase enhances the breakdown and assimilation of phosphorus, ultimately reducing the excretion of phosphorus into the environment [18,19,20]. Therefore, supplementations of phytase improves growth and feed efficiency [21]. Furthermore, corn cob (CC), wheat straw (WS) and sugarcane bagasse (SCB) are major agricultural by-products in Pakistan that can be easily processed to biochar and fed to poultry. To our current understanding, no study has investigated the effect of biochar supplementation of CC, WS and SCB in diets containing phytase in broilers on growth performance, nutrient digestibility, carcass yield, bone mineralization, litter quality and footpad lesions in broilers. Therefore, the purpose of the present research is to determine the effect of CC, WS and SCB biochar supplementation on growth performance, nutrient digestibility, carcass yield, bone mineralization, litter quality and footpad lesions in broilers. The current experiment hypothesizes is that supplementation of 1% biochar from different agricultural sources, in the presence of phytase, will improve growth performance, nutrient digestibility, bone mineralization, immune response and litter quality in broilers, with a comparison of the effectiveness of each biochar type.

## 2. Materials and Methods

The research trial was conducted at the Research and Development Farm, Mukhtar Feeds, Samundri. All the procedures carried out in the current experiment were approved by the Director, Graduate Studies (IADS/2021/124 and IADS/2021/132).

### 2.1. Preparation of Biochars

Biochars were prepared through slow pyrolysis in a portable metallic kiln CCM-7 (Eco Research Ltd., Birmingham, UK), a UK-designed carbon catcher model with a capacity of 10 kg. Biochars were prepared batch by batch at the University of Agriculture, Faisalabad, by burning the raw materials inside the kiln in an anaerobic or oxygen-limited environment at 450–550 °C for 120–150 min, following Keba et al. [22]. The production process yielded biochar equivalent to 18–25% of the input biomass.

### 2.2. Experimental Birds and Plan

A total of eight hundred (800) day-old broiler chicks were randomly divided into four treatments, having 10 replicates per treatment (20 chicks/replicate). Upon arrival, chicks were weighed individually, with an average body weight of 39 ± 2 g. The house temperature was maintained at 95 °F for the first week and then gradually reduced by 5 °F each week until it reached 75 °F. Chicks in each replicate were kept in separate pens 1.83 m × 1.22 m × 0.76 m (length, width and height, respectively). A 5.08 cm deep layer of rice husk was used as a bedding material on the floor and eventually spread throughout the pens. The chicks were subjected to 24 h light availability for easy access to feed and water ad libitum, following Kamal et al. [23] and Liaqat et al. [24].

### 2.3. Experimental Diets

A 35-day experiment was conducted under a completely randomized design. Three diets for the starter (0–7 days), grower (8–21 days) and finisher (22–35 days) phases of broilers were formulated and presented in Table 1. The study included four treatments: a basal diet acted as control, basal diet supplemented with CC biochar (1%), basal diet supplemented with WS biochar (1%) and a basal diet supplemented with SCB biochar (1%). An inclusion level of 1% was chosen in accordance with earlier studies that evaluated similar levels and reported favorable outcomes on broiler performance and health parameters without adverse effects [14,25]. The experimental diets were also supplemented with phytase enzymes.

The methods highlighted by Naveed et al. [26] were followed to analyze the ingredients of the experimental diet for dry matter (DM), crude protein (CP) and ether extract (EE) content before feed formulation. Phytase was supplemented at an inclusion rate of 0.02% (i.e., 200 g/ton of feed or 2,000,000 FTU/ton), and dietary specifications for the birds were maintained at standard values as outlined by the NRC throughout the starter, grower and finisher phases.

### 2.4. Feed Intake and Growth Performance

The research design followed earlier studies to compute FI measurements and growth performance figures, including BWG and FCR weekly. The FI measurement occurred through a difference method, which determined the input feed against the rejected portions. The weekly FI measurement per bird requires division of the replicate total intake by the total bird count within each replicate. The daily bird mortality rates were also noted daily to compute corrected FI and detect any systematic errors. The birds’ BWG, like the FI, was calculated weekly. To proceed with this, chicks were weighed initially to record body weight on the 1st day, and birds per replicate were then weighed weekly. As with the FI measurements, the difference approach was utilized to calculate the BWG weekly. As a result, the weekly BWG was determined by subtracting the birds’ initial body weight at the beginning of the week from their final body weight after the week. The FCR was calculated by dividing the feed consumed per bird over a specific period by the weight gained during that period.

### 2.5. Nutrient Digestibility

The digestibility of the nutrient was determined on the 21st and 35th day of the experimental trial using the Celite (acid-insoluble ash) as an indirect marker. Celite^®^ (Celite Corp., Lompoc, CA, USA) was introduced to the broilers’ experimental diets until the 35th day. For the evaluation of ileal nutritional digestibility, four birds per pen were picked randomly and slaughtered via cervical dislocation. Digestibility coefficients reported in this study represent apparent ileal digestibility, calculated from the collected ileal contents. The ileum area of the small intestine is demarcated from Meckel’s diverticulum to 40 mm proximal to the ileocecal junction. A few drops of formalin solution were added to the 200 mL plastic cup, in which the ileal contents were flushed out, to prevent all microbial activity, following Udoumoh et al. [27]. The obtained fecal samples were moved to the laboratory for additional evaluation. Digestibility values represent apparent ileal digestibility, as determined by ileal content collection and acid-insoluble ash as a marker, following the protocol of Liaqat et al. [24].

### 2.6. Carcass Yield and Lymphatic Organs and Antibody Titers

To evaluate different carcass characteristics, such as live bird weight, breast meat and thigh percentages, relative weights of the gizzard, liver and heart, as well as dressing percentage, measurements were performed on two randomly selected birds from each replicate on the 21st and 35th days of the trial. The weight of immune organs, including thymus, spleen and bursa, was measured after slaughter for determining relative organ weight percentages through the following formula: (organ weight/carcass weight) × 100. The data of lymphatic organs were also recorded after removing the adherent tissues on the 35th day of experiment. Antibody titers for Newcastle disease (ND) virus were measured using the heme-agglutination inhibition technique, and serum collected for infectious bronchitis virus (IBV) titers were measured by a commercial ELISA kit.

### 2.7. Bone Mineralization and Footpad Lesions

At the end of the experiment, two birds per replicate were dissected following Kim et al. [28]. Both right and left tibias of the broilers were removed. The right tibias were dried at 100 °C for 24 h after being cleaned. Dried tibias were then extracted with ethyl ether in a Soxhlet apparatus for 48 h, dried again and ashed to measure tibia Ca and P concentrations by inductively coupled plasma spectrometer (Optima 5300 DV, Perkin Elmer Inc., Shelton, CT, USA) as demonstrated by Kurtoğlu et al. [29] and Watson et al. [30]. On the other hand, the left tibias were subjected to breaking strength analysis utilizing a TA-HDi texture analyzer (Stable Micro Systems Ltd., Godalming, UK) as described by Shaw et al. [31]. After the trial, litter samples were collected from three key areas of each pen, i.e., near the water source, near the feeder and the central area. Litter quality was evaluated by collecting samples from three locations in each pen (feeder, water source and center). Samples were analyzed for moisture content, nitrogen content and pH as described by Pope and Cherry [32]. Furthermore, footpad lesions were assessed in randomly selected four birds per replicate. Scoring was performed utilizing a six-point visual scale: 0 = healthy footpad without lesions; 1 = slight discoloration or minor surface damage; 2 = dark papillae without ulceration; 3 = small ulceration with scab formation; 4 = moderate ulceration with crust and 5 = extensive ulcer covered with crust. The six-point footpad scoring system used was based on validated criteria by Michel et al. [33] and Piller et al. [34] to ensure reproducibility.

### 2.8. Chemical Analysis

For the chemical analysis of feed and feces, individual samples from each replicate were pooled together to form eight composite samples for each treatment. A hot air oven (Heraeus, Hanau, Germany), operated at 65 °C, was used to dry the samples before passing through a sieve of 0.5 mm diameter for powdering. Predetermined standard methods were used to examine DM and evaluate CP levels and EE content before subjecting ground samples to a −20 °C temperature storage. Measuring nitrogen content with the Kjeldahl method enabled the calculation of CP through multiplication of nitrogen content by a factor of 6.25. Ether extract of the feed sample and the collected feces was analyzed through the Soxhlet apparatus following Sangsopha et al. [35]. The ash samples extracted from both feed and feces were used for the determination of acid-insoluble ash. Apparent nutrient digestibility was calculated following Liaqat et al. [24].

### 2.9. Chemical Characterization of Biochar

After the pyrolysis process, all biochar samples were ground and sieved through a mesh with openings smaller than 0.5 mm. Proximate and mineral analyses, as mentioned in Table 2, were performed in duplicate to characterize the biochar. For proximate composition, moisture content was analyzed by drying the samples in a hot air oven at 105 °C. Volatile matter content was determined by combusting 1 g of biochar in a lidded crucible at 950 °C for 11 min, while ash content was assessed by burning the sample in an uncovered crucible at 750 °C for two hours. Fixed carbon content was calculated by subtracting the sum of moisture, ash and volatile matter percentages from 100% [36]. For mineral analysis, powdered samples of known weight were incinerated at 760 °C in a muffle furnace for 6 h, and the resulting ash was digested in HCl, diluted with deionized water and analyzed. Sodium and potassium contents were quantified using a flame photometer, while magnesium, manganese, zinc and copper were measured by atomic absorption spectrophotometry. Phosphorus was determined by spectrophotometry according to Cantrell et al. [37].

### 2.10. Statistical Analysis

An arcsine transformation was employed to process mortality percentages before the statistical evaluation. The statistical software Minitab 17 applied one-way ANOVA analysis under the Completely Randomized Design to evaluate the collected data. Tukey’s post hoc test revealed the statistically significant differences that existed among the treatment groups. Any values of *p* less than 0.05 determined statistical differences between groups under assessment.

## 3. Results

### 3.1. Growth Performance

The effect of dietary supplementation of biochars (CC, WS and SCB biochar) on the growth performance of broilers during different phases (starter, grower and finisher) is shown in Table 3. In the starter phase and grower phases, significant differences (*p* < 0.05) in FI and BWG were found between the treatments, with the lowest being in CC biochar. Similarly, in the finisher phase, significant differences (*p* < 0.05) were observed as well in FI and BWG, with the highest FI in the control and the highest BWG in WS biochar. In the starter, grower and overall phases, significant differences (*p* < 0.05) were observed in FCR, with better FCR in CC biochar, while in the finisher phase, WS biochar showed better results.

### 3.2. Nutrient Digestibility

The results regarding dietary supplementation of biochars on nutrient digestibility are displayed in Table 4. Results indicated that on the 21st day, significant differences (*p* < 0.05) were found in the digestibility of DM, CP and EE. The maximum DM and CP digestibility were noted in WS biochar and SCB biochar, respectively, compared to other treatments. Similarly, the maximum EE digestibility was assessed in SCB biochar, with the least observed in the control group. Furthermore, the nutrient digestibility on the 35th day continued to differ significantly between treatments. The highest nutrient digestibility (DM, EE and CP) was shown in SCB biochar, while the lowest was observed in the control.

### 3.3. Carcass Characteristics

The effect of biochar supplementation on carcass characteristics and giblet parameters of broilers at days 21 and 35 is indicated in Table 5. On the 21st and 35th day of analysis, no significant differences (*p* > 0.05) were found between treatments for carcass characteristics. On the 21st day, the control group showed numerically the highest carcass and breast percentage, while SCB biochar had the lowest carcass and thigh percentage. Similarly, giblet organs (liver, heart and gizzard) did not show significant differences between treatments. However, liver and gizzard percentages were numerically highest in the control group. On the 35th day, SCB biochar resulted in the highest carcass and breast percentages, although these differences were not statistically significant (*p* > 0.05).

### 3.4. Bone Mineralization, Litter and Footpad Quality

The results regarding the effect of biochar supplementation on bone mineralization, foot score and litter score of broilers on the 21st and 35th day are shown in Table 6. Significant differences (*p* < 0.05) were observed in bone P and Ca percentages, with the highest percentages observed in WS biochar on the 21st day. The analysis of the 35th day indicated that SCB biochar had the highest Ca percentage and the highest P percentage in WS biochar. Moreover, significant differences (*p* < 0.05) were observed in litter quality and food pad score, with the highest score shown in the control group on both the 21st and 35th days.

### 3.5. Lymphoid Organs (%) and Antibody Titer of Newcastle Disease and Infectious Bronchitis

The average relative weights of lymphoid and antibody titer of Newcastle disease and infectious bronchitis in broilers as affected by dietary treatments are shown in Table 7. Obtained data revealed that the relative weight of the spleen was not affected by biochar supplementation in diet, while dietary supplementation of CS and WS biochar in the diet resulted in the highest relative weights of thymus and bursa (*p* < 0.05). However, dietary supplementation of WS, SC and SCB biochars significantly increased antibody titers, with the greatest improvement observed in the SCB group, where ND virus titers increased by approximately 7% and IBV titers by around 6.7% compared to the control group (*p* < 0.05).

## 4. Discussion

Agricultural and livestock production are critical to food security, but they generate substantial organic waste that poses environmental challenges [38]. However, agricultural and livestock waste are increasingly being recycled [39,40], and agricultural residues are used as organic fertilizers to enhance soil fertility [41], while livestock by-products and agro-industrial waste are processed into valuable feed ingredients [42,43,44]. These sustainable practices not only reduce pollution [45,46] but also improve animal health [47,48,49] and productivity [50,51,52,53], contributing to circular agriculture and resource efficiency [41,54]. Corn cob, WS and SCB are also some of the agriculture by-products that are being used as biochar in animal feed to improve the feed efficiency, growth, and immunity. In this study, supplementation of various biochars (CC, WS and SCB) resulted in a reduction in FI in broilers during the finisher phase. These findings are consistent with previous research by Jindal et al. [55], Kana et al. [25] and Dim et al. [12], who also reported reduced FI with biochar inclusion. The increase in feed bulk density of biochar results in longer intestinal retention time for feed, which decreases its palatability across the digestive system. Research carried out by Odunsi et al. [56] and Prasai et al. [57] contradicts these findings, demonstrating increased FI when biochar levels increased, likely due to the adsorbent qualities of biochar that decrease gastrointestinal surface tension, leading to better nutrient uptake [58]. In contrast, the research findings of Prasai et al. [59] and Sung et al. [60] did not show any variations in FI levels after biochar addition. While statistical differences in BWG and FCR were observed, broilers supplemented with biochar, particularly WS and CC, showed up to a 4.7 percent increase in BWG and a 6.7 percent improvement in FCR compared to the control group. These improvements reflect meaningful enhancements in production efficiency, potentially contributing to better overall flock performance and resource utilization in commercial broiler systems.

Results from this study showed that biochars enhanced the nutrient digestibility (CP, EE and DM) of broiler birds. These results are in accordance with the findings of Evans et al. [15], who showed that biochar binds toxins and harmful substances, resulting in better nutrient digestibility by enhancing the digestive process. Likely, the slower passage rate from the intestinal tract enhanced the effect of nutrient absorption [61]. However, contrary results were found by Jiya et al. [62], who reported no effect on nutrient digestibility by supplementation of biochar. The presence of phytase in all treatment diets may have enhanced phosphorus availability, and its combination with biochar could have contributed to improved mineral utilization. This potential interaction warrants further investigation to determine whether biochar enhances the efficacy of phytase in broiler diets.

The present investigation demonstrated that supplementation of biochars (CC, WS and SCB) at 1% improved BWG. This outcome is in agreement with the results of Kana et al. [25] and Majewska et al. [63], who documented a rise in BWG in broilers fed diets with biochar. Biochar’s potential to capture toxic compounds and anti-nutritional agents helps explain the increased growth of birds, which could otherwise impair nutrient absorption in the digestive system [57]. However, the research by Kalus et al. [13] and Odunsi et al. [56] suggests that BWG declines at increased levels (4–5%) of biochar supplementation, indicating that high biochar amounts might be harmful. The findings of Prasai et al. [59] show that arsenic in poultry manure biochar led to a reduction in BWG as it negatively impacted the digestive system and growth performance.

The results indicated that FCR improved with the supplementation of various biochars (CC, WS and SCB) at 1%. This observation is consistent with previous research by Kana et al. [25] and Prasai et al. [64], who reported enhanced FCR with biochar supplementation as a result of a slower transit time in the gastrointestinal tract, leading to better feed utilization. However, Sung et al. [60] and Evans et al. [11] found no significant effect on FCR with biochar supplementation.

Furthermore, the current study revealed that introducing 1% of biochars consisting of CC, WS and SCB improved FCR. The research findings supported previous results from Prasai et al. [64], who discovered enhanced FCR through biochar supplementation as the gastrointestinal tract allowed reduced feed passage and enhanced feed utilization. However, Evans et al. [11] determined that biochar addition did not produce any noteworthy impact on FCR.

The study showed that adding different types of biochar in amounts up to 1% did not produce significant modifications to carcass parameters. Studies by Kutlu et al. [58] demonstrated similar results since biochar supplementation showed no impact on carcass characteristics. Kana et al. [25] exhibited that levels around 1% might be below the threshold needed to elicit significant changes in carcass parameters. However, several studies, like Mohammed and Billa [65] demonstrated that higher biochar supplement levels resulted in improved carcass yield with increased weights in breast muscle and thigh and wings. In our study, although differences were not statistically significant, broilers receiving SCB biochar showed numerically higher carcass and breast percentages on day 35, indicating a possible biological effect that merits further investigation.

The inclusion of biochar in the diet positively influenced bone mineralization, specifically tibia bone development, aligning with the study results presented by Evans et al. [11]. A higher amount of poultry litter biochar resulted in better tibia quality because it increased the accessibility of essential minerals, including calcium and phosphorus. On the contrary, Safaeikatouli et al. [61] discovered that bone mineralization stayed unchanged with different biochar sources, indicating that dosage and source of biochar influence the outcome.

The current study showed that biochar supplementation improved litter quality, likely due to its water-absorbing properties, which reduced moisture in the excreta. These findings are in line with Linhoss et al. [1] and Hinz et al. [66], who reported better litter quality and improved footpad health due to biochar supplementation. In our study, footpad lesions were significantly lower in the birds fed with biochar, particularly with CC biochar, which also improved overall litter quality. This suggests that biochar’s high water absorption capacity may enhance both footpad health and litter conditions in broiler production.

Moreover, incorporating biochar into the diet increased litter quality through its water-absorption capacity, ultimately reducing the moisture levels in the excreta. The findings of this study are corroborated by Linhoss et al. [1], who established that incorporating biochar in poultry diets improves the quality of litter and footpad health in animals. In our study, the inclusion of biochar in the diet was associated with fewer footpad lesions while providing beneficial effects on the overall quality of the litter, especially using CC biochar. This suggests that biochar’s capability to absorb high amounts of water helps improve footpad health in addition to enhancing overall litter conditions in broiler farming.

While this study did not analyze the arsenic level of the biochars, the sources used (CC, WS and SCB) were clean, plant-based materials and the pyrolysis conditions (450–550 °C) were similar to those used in other broiler studies [14,25], where similar biochars were supplemented at 1% without assessing arsenic levels and no negative effects were reported. Moreover, Evans et al. [15] showed that broiler performance was unaffected by arsenic concentrations in poultry litter biochar below 22 ppm and that growth reduction only happened at concentrations close to 99 ppm. Because of this precedent and the comparative nature of our trial, an analysis of arsenic was not carried out. However, the importance of elemental profiling, including arsenic analysis, is recognized, and such evaluations will be incorporated in future studies.

From data obtained in our study, it is clear that biochar supplementation in the diet significantly increased antibody titers, which may be partially associated with reduced ammonia exposure due to improved litter quality. High ammonia levels are known to impair immune function in poultry by decreasing antibody titers like NDV and IBV [67]. A previous study has reported that antibody titer was significantly reduced at 21st day in broilers when ammonia levels reached 26 ppm or over that level. It is also well documented that high levels of antibody titers against Newcastle disease may be related to the improvement of bursa Fabricius as an important lymphoid organ.

## 5. Conclusions

The supplementation of 1% biochar derived from CC, WS and SCB in broiler diets showed notable enhancement in growth performance, bone mineralization, footpad condition, litter quality, lymphoid organs and immunity, having no significant impacts on carcass characteristics. Among the sources tested, SCB biochar produced the highest antibody titers and breast yield, WS biochar was more effective in enhancing thymus and bursa weight and bone mineral content, while CC biochar showed better effects on footpad condition and nutrient digestibility. Although improvements in both litter quality and immunity were observed, the causal relationship between these outcomes remains to be fully elucidated. The findings indicate that adding dietary supplementation with biochar proves to be a valuable inclusion in animal nutrition, offering practical benefits for poultry health and sustainable poultry management practices. However, further research is required to establish ideal supplementation rates and long-term effects in poultry nutrition. Subsequent studies examining the underlying mechanisms behind biochar’s influence on gut health and immune response would provide deeper insights.

## Figures and Tables

**Table 1:** Diet and nutrient composition for starter, grower and finisher phases.

Ingredients	Starter (0–7 Days)	Grower (8–21 Days)	Finisher (22–35 Days)
Maize	52.57	60.76	62.20
Soybean meal	28.51	26.92	25.00
Canola meal	7.45	5.00	5.10
Rice polish	4.99	2.99	2.79
Poultry by-product meal	3.00	1.23	1.84
Phytase (10,000 FTU/g)	0.02	0.02	0.02
NaHCO_3_	0.98	0.77	0.88
Monocalcium phosphate	0.80	0.52	0.76
Lysine sulphate 55%	0.45	0.42	0.38
DL-Methionine 99.5%	0.32	0.31	0.29
NaCl	0.42	0.51	0.31
^1^ Premix	0.24	0.28	0.24
L-Threonine 99%	0.21	0.24	0.14
L-Valine 99%	0.04	0.03	0.06
Total	100	100	100
**Nutrient Composition**			
Crude protein	23.00	21.00	19.00
^2^ ME (kcal/kg)	3000	3100	3200
Calcium	0.96	0.96	0.79
Available phosphorus	0.48	0.48	0.39
Sodium	0.19	0.20	0.18
Chloride	0.17	0.19	0.18
Digestible lysine	1.28	1.15	1.08
Digestible methionine	0.56	0.51	0.47
^3^ Digestible M + C	1.08	0.99	0.91
Digestible valine	1.10	1.00	0.90
Digestible isoleucine	0.97	0.89	0.81
Digestible arginine	1.52	1.37	1.22
Digestible tryptophan	0.23	0.21	0.19
Digestible threonine	0.97	0.88	0.78

^1^ Each kg of premix was provided per kg of diet: 10,000 IU vitamin A, 1100 IU vitamin D_3_, 11.0 IU vitamin E, 1.1 mg vitamin K, 2.2 mg Thiamin, 5 mg Riboflavin, 12 mg Pantothenate, 2.2 mg vitamin B_6_, 0.11 mg d-biotin, 1.55 mg Folic acid, 12.1 µg vitamin B_12_, 250 mg Choline chloride, 44 mg Nicotinic acid, 50 mg Zn, 60 mg Mn, 5 mg Cu, 0.1 mg Co, 0.3 mg I, 30 mg Fe and 1 mg Se; ^2^ Metabolizable energy; ^3^ Methionine + Cysteine.

**Table 2:** Chemical composition and mineral content of biochars derived from wheat straw, corncob and sugarcane bagasse.

Parameter	Wheatstraw Biochar	Corncob Biochar	Sugarcane Bagasse Biochar
Ash (%)	20	21	22
Volatile matter (%)	23	19	22
Moisture (%)	3	3.4	4.5
Fixed carbon (%)	54	56.6	53.5
Zinc (mg/L)	13.8	13.4	24.4
Manganese (mg/L)	14.6	13.3	17.2
Copper (mg/L)	25.7	21.38	27.32
Magnesium (mg/L)	165.5	163.3	141.4
Phosphorus (mg/100 g)	220	350	320
Sodium (mg/100 g)	50	70	50
Potassium (mg/100 g)	40	30	32

**Table 3:** Impact of various biochars on growth performance during starter, grower, finisher and overall phases of broilers.

Parameters	Treatments				SEM	*p*-Value
	Control	Corn Cob Biochar (1%)	Wheat Straw Biochar (1%)	Sugarcane Bagasse Biochar (1%)		
**Starter Phase (0–7 days)**						
^1^ FI	126.63 ^a^	123.25 ^c^	126.00 ^ab^	124.75 ^b^	0.342	0.001
^2^ BWG	92.62 ^b^	99.62 ^a^	99.75 ^a^	98.75 ^a^	1.34	0.021
^3^ FCR	1.36 ^a^	1.23 ^b^	1.25 ^b^	1.26 ^b^	0.01	0.018
**Grower Phase (8–21 days)**						
FI	1040.01 ^a^	1002.94 ^b^	1007.92 ^b^	1005.43 ^b^	5.60	0.001
BWG	723.66 ^b^	792.88 ^a^	766.99 ^a^	771.10 ^a^	10.75	0.026
FCR	1.41 ^a^	1.26 ^b^	1.31 ^b^	1.30 ^b^	0.01	0.001
**Finisher Phase (22–35 days)**						
FI	1803.41 ^a^	1785.33 ^b^	1784.20 ^b^	1789.21 ^b^	2.88	0.002
BWG	1170.10 ^c^	1187.21 ^b^	1201.14 ^a^	1196.54 ^a^	3.13	0.001
FCR	1.54 ^a^	1.50 ^b^	1.48 ^b^	1.49 ^b^	4.27	0.001
**Overall Phase (0–35 days)**						
FI	2970.05 ^a^	2911.52 ^b^	2918.12 ^b^	2919.39 ^b^	1.02	0.031
BWG	1986.38 ^b^	2079.71 ^a^	2067.88 ^a^	2066.39 ^a^	1.11	0.009
FCR	1.49 ^a^	1.39 ^b^	1.41 ^b^	1.41 ^b^	0.02	0.012

^1^ Feed intake; ^2^ Body weight gain; ^3^ Feed conversion ratio; SEM: Standard error; different superscripts in a row indicate *p* < 0.05.

**Table 4:** Impact of various biochars on nutrient digestibility of broilers on the 21st and 35th day.

Digestibility Percentage		Treatment			SEM	*p*-Value
	Control	Corn Cob Biochar (1%)	Wheat Straw Biochar (1%)	Sugarcane Bagasse Biochar (1%)		
**On 21st day**						
Dry matter	79.65 ^b^	84.06 ^a^	85.92 ^a^	84.84 ^a^	0.459	0.002
Ether extract	78.03 ^b^	82.56 ^ab^	84.92 ^a^	85.34 ^a^	0.783	0.014
Crude protein	79.82 ^b^	84.07 ^ab^	84.37 ^ab^	85.50 ^a^	0.824	0.029
**On 35th day**						
Dry matter	79.82 ^c^	83.07 ^b^	84.36 ^ab^	85.50 ^a^	0.875	0.037
Ether extract	78.53 ^c^	82.06 ^b^	83.92 ^ab^	84.84 ^a^	0.716	0.011
Crude protein	80.65 ^b^	83.57 ^ab^	83.83 ^ab^	86.42 ^a^	0.814	0.029

SEM: Standard error; different superscripts in a row indicate *p* < 0.05.

**Table 5:** Impact of various biochars on carcass yield of broilers on 21st and 35th day.

Parameters (%)	Treatments				SEM	*p*-Value
	Control	Corn Cob Biochar (1%)	Wheat Straw Biochar (1%)	Sugarcane Bagasse Biochar (1%)		
**On 21st day**						
Dressing	95.93	95.72	96.78	97.35	0.428	0.149
Carcass	59.37	56.22	55.44	54.42	1.469	0.490
Breast	30.29	29.99	27.46	28.93	1.262	0.469
Thigh	22.72	25.06	22.69	22.57	1.155	0.392
Heart	0.50	0.61	0.62	0.74	0.044	0.79
Liver	3.51	3.22	3.16	3.35	0.233	0.74
Gizzard	2.88	2.24	2.49	2.54	0.196	0.29
**On 35th day**						
Dressing	98.11	96.64	97.35	97.22	0.778	0.646
Carcass	60.08	60.90	59.93	61.22	1.394	0.891
Breast	31.82	32.33	32.67	34.17	0.882	0.375
Thigh	24.32	25.03	24.06	23.68	0.833	0.723
Heart	0.38	0.64	0.44	0.47	0.067	0.188
Liver	2.49	2.25	2.65	2.32	0.295	0.782
Gizzard	1.19	1.32	1.60	1.36	0.147	0.383

SEM: Standard error.

**Table 6:** Impact of various biochars on bone mineralization, foot score and liter score of broilers on 21st and 35th day.

Parameters (%)	Treatment				SEM	*p*-Value
	Control	Corn Cob Biochar (1%)	Wheat Straw Biochar (1%)	Sugarcane Bagasse Biochar (1%)		
**On 21st day**						
Bone P	18.18 ^b^	22.08 ^ab^	27.22 ^a^	22.66 ^ab^	1.27	0.033
Bone Ca	4.60 ^b^	5.70 ^a^	5.72 ^a^	5.30 ^ab^	0.17	0.027
Foot Score	1.00 ^a^	0.12 ^b^	0.25 ^b^	0.37 ^ab^	0.16	0.004
Liter Score	1.87 ^a^	1.12 ^b^	1.0 ^b^	1.12 ^b^	0.15	0.002
**On 35th day**						
Bone P	16.66 ^d^	19.56 ^d^	25.31 ^a^	17.72 ^c^	0.05	0.004
Bone Ca	4.50 ^d^	5.32 ^b^	4.84 ^c^	5.74 ^a^	0.04	0.001
Foot Score	0.87 ^a^	0.12 ^b^	0.25 ^ab^	0.25 ^ab^	0.17	0.021
Liter Score	1.75 ^a^	1.12 ^b^	1.00 ^b^	1.12 ^b^	0.15	0.007

SEM: Standard error; different superscripts in a row indicate *p* < 0.05.

**Table 7:** Impact of various biochars on relative weight of lymphoid organs (%), antibody titer of Newcastle disease and infectious bronchitis in broilers.

Parameters (%)	Treatment				SEM	*p*-Value
	Control	Corn Cob Biochar (1%)	Wheat Straw Biochar (1%)	Sugarcane Bagasse Biochar (1%)		
**Lymphoid organs (%)**						
Spleen	0.07	0.08	0.10	0.09	0.071	0.083
Thymus	0.12 ^b^	0.17 ^a^	0.17 ^a^	0.14 ^b^	0.007	0.027
Bursa	0.09 ^b^	0.11 ^a^	0.12 ^a^	0.10 ^ab^	0.001	0.044
**Serum antibody levels**						
^1^ ND (Log_2_)	8.7 ^b^	9.2 ^a^	9.0 ^ab^	9.3 ^a^	0.02	0.004
^2^ IBV	1499 ^b^	1553 ^ab^	1564 ^ab^	1600 ^a^	0.032	0.001

^1^ ND: Newcastle disease, ^2^ IBV: Infectious bronchitis virus, SEM: Standard error of means based on 10 replicates, Means within a row with (^a^, ^ab^, ^b^) superscripts differ significantly (*p* < 0.05).

## Data Availability

Data will be available by corresponding authors.
